# “Driver-passenger” bacteria and their metabolites in the pathogenesis of colorectal cancer

**DOI:** 10.1080/19490976.2021.1941710

**Published:** 2021-07-05

**Authors:** Marion Avril, R. William DePaolo

**Affiliations:** a Department of Medicine, University of Washington, Seattle, WA, USA; b Department of Medicine, Center for Microbiome Sciences & Therapeutics, University of Washington, Seattle, WA, USA

**Keywords:** Colorectal cancer, microbiome, driver-passenger, metabolites, mucus, biofilm

## Abstract

Colorectal cancer (CRC) is a significant public health problem accounting for about 10% of all new cancer cases globally. Though genetic and epigenetic factors influence CRC, the gut microbiota acts as a significant component of the disease’s etiology. Further research is still needed to clarify the specific roles and identify more bacteria related to CRC development. This review aims to provide an overview of the “driver-passenger” model of CRC. The colonization and active invasion of the “driver(s)” bacteria cause damages allowing other commensals, known as “passengers,” or their by-products, i.e., metabolites, to pass through the epithelium . This review will not only focus on the species of bacteria implicated in this model but also on their biological functions implicated in the occurrence of CRC, such as forming biofilms, mucus, penetration and production of enterotoxins and genotoxins.

## Introduction

Globally, colorectal cancer (CRC) is the third most common cause of cancer mortality accounting for approximately 1.2 million new cases and 600,000 deaths per year^1^. The etiology of CRC is still not fully understood. While host genetics account for a small proportion of disease, lifestyle habits and environmental exposures comprise most cases. Epidemiological data that show an increased risk of CRC in populations that migrate to Western countries or adopt their lifestyle,^2^ suggesting that CRC is a disease whose etiology is defined primarily by interactions between the host and its environment. Established environmental factors include alcohol consumption,^3,4^ lack of physical activity,^4^ smoking,^5^ obesity,^5^ and diet.^2^

Interestingly, the gut microbiome sits directly at the interface between environment and host and has been recognized as a modulator of CRC.^6^ A collection of 100–400 × 10^12^ bacteria lives in a mutualistic relationship with the human cells of the gastrointestinal (GI) tract.^7^ Collectively this community of bacteria, viruses, fungi, protists, and their collective genomes are referred to as the microbiome. In a ratio equal to our human cells but outnumbering our genes by a factor of 100,^8,9^ the microbiome is considered as a virtual organ that participates in the physiological functioning throughout the host’s life. In the gut, the microbiota participates in digestion, wards off pathogens, and primes the immune system. The human microbiome is initially transmitted through the mother during childbirth. A recent longitudinal study analyzing the microbiome from 596 full-term babies born in UK hospitals demonstrated that the mode of delivery (vaginal vs. cesarean section) is a significant factor that affects the composition of their gut microbiota from their neonatal period into infancy.^10^ Indeed, vaginally delivered babies have a microbiome that is mostly matched the mother’s gut and reproductive tracts compared to the babies born via cesarean section who had many fewer of these bacteria.^10^

Immediately after birth, newborn babies experience a rapid colonization by their mother’s microbiota while other bacteria such as *Bifidobacteria* and *Lactobacillus* are acquired during early life. A lack of exposure to the “right” commensal species in early childhood has been associated with asthma, allergies, and diabetes.^11^ As the child grows, the addition of solid food drives the complexity of bacterial communities. Consequently, more than 500 different species may be present in the commensal microbiota of normal adult intestines^12^ composed of facultative anaerobes (*Lactobacilli, Streptococci Enterococci* and *Enterobacteria*), and strict anaerobes (*Bifidobacterium, Bacteroides, Eubacterium, Fusobacterium, Peptostreptococcus*, and *Atopobium)*.^13^ The majority of microbial species found in the gut of healthy individuals belong to *Firmicutes* (>70%), Bacteroidetes (>30%), *Proteobacteria* (<5%), *Actinobacteria* (<2%), *Fusobacteria* and *Verrucomicrobia* (<1%), and other phyla.

While those bacteria are permanent residents and considered symbionts, helping to maintain homeostasis, during a lifetime they may become pathogenic due to the genetic background of the host and/or exposure to environmental and lifestyle factors. Repeated exposure to environmental stressors may eventually lead to a permanent shift in the overall composition of the microbiota. This shift in composition is referred to as dysbiosis and has been associated with several chronic inflammatory diseases. It has been shown that the changes in the ratio of two phyla, *Firmicutes* and *Bacteroidetes*, are associated with Inflammatory Bowel Disease (IBD)^14^ and obesity,^15^ both risk factors for CRC.^16^ Local inflammation caused by disease can further contribute to changes in the microbiota. For instance, as a tumor develops, this may lead to changes in the intestinal barrier allowing passage of commensals and toxins into the underlying tissue, altering the intestinal microenvironment. Some bacteria will be able to thrive in the new intestinal milieu, while others will be unable to subsist and will decrease in abundance or even disappear.

An interesting way to explain the role of microorganisms in CRC development was proposed by Tjalsma and colleagues in the “bacterial driver-passenger” model.^17^ This model states that the initiation of CRC is triggered by local mucosal colonization by specific pathogens (drivers). These driver bacteria cause changes in the tumor microenvironment allowing for colonization by opportunistic (passengers) bacteria that facilitate disease progression. Nevertheless, the initiation and progression of CRC is not due to a unique microorganism but to several species identified as contributors to this pathogenesis.^18^ Several commensal bacteria have been classified as pathogens associated with cancer including, *Streptococcus bovis, Helicobacter pylori, Fusobacterium nucleatum, Bacteroides fragilis* and *Enterococcus faecalis*. While others such as *Lactobacillus acidophilus* and *Bifidobacterium longum*, may inhibit colorectal tumorigenesis.^19^ Each bacterium contributes to carcinogenesis by a distinct microbial signature, such as the production of deleterious metabolites or by-products, the stimulation or inhibition of local immune responses, or modulation in gene expression.

In this review, we focus our current knowledge on the role of the different microbial species involved in these “driver-passenger” models that engage this mutualistic relationship with the GI tract, as well as the potential role of their microbial by-products in regulating CRC. With this purpose, a systematic literature review from the electronic databases PubMed and Embase for studies of the last 10 years, has been carried out. Searches were conducted using the terms “colorectal cancer or CRC” and “driver-passenger” or “microbiota” or “gut” or “metabolites”. Relevant studies were also identified through manual searches of reference lists of selected studies and recently published review articles. We intentionally did not include any mechanism of immunogenicity of neoantigens in CRC in this review. This topic was intensively reviewed recently by Sillo and colleagues,^20^ where neoantigens is a result of non‐synonymous somatic mutations during tumor evolution, also known as driver mutations, which cause the transformations required for tumorigenesis and tumor propagation.

## Current models explaining the role of the microbiota in CRC

### The ‘Alpha-Bug’ model

The ‘Alpha-bugs’ were defined by Yu and Fang^19^ as certain intestinal commensal bacteria that produce epithelial gene mutations directly or indirectly. Alexander et al.^21^ postulate that the initiation of early neoplastic lesions may be due to pathogenic bacteria colonizing genetically susceptible people which can lead to inflammation, cell proliferation, and production of genotoxins substrates. But at the core of the models is that a single bacterium is linked with tumorigenesis. For example, in the 1980s, *Helicobacter pylori* was identified in the stomach of patients with gastritis and peptic ulceration, providing a link between this bacterium and gastric cancer.^22^ In 2010, Sears and colleagues^23,24^ proposed the ‘Alpha-bug’ model by defining the capacity of ETBF to induce adenoma tumors in APC (Min/+) mice. These *in vitro* studies demonstrated the mechanism of action of *B. fragilis* toxin BFT (a 20-kDa zinc-dependent metalloprotease toxin) was to cleave E-cadherin, causing an increase in colonic permeability, and translocation of bacterial antigens. The activation of the intestinal immune cells by these bacterial antigens is thought to be one factor contributing to the induction of colonic inflammation in IBD, a known precursor of intestinal cancer. Another “alpha-bug” is *Escherichia coli*, which has been found associated with the mucosa in CRC.^25^ *E. coli* can promote tumor growth, *in vitro* and *in vivo*, via the production of colibactin^26^ as well as other oncogenes. *E. coli* strains harboring the pks island (pks+ *E. coli*) have also been found in CRC and have a carcinogenic effect independent of inflammation in an AOM/IL-10^−/-^ (azoxymethane/interleukin) mouse model of CRC. A third alpha-bug was demonstrated by Kostic et al.,^27,28^ who identified an enrichment of *Fusobacterium* spp. in human colonic adenomas and stool samples from colorectal adenoma and carcinoma patients. Additionally, they demonstrated that Apc (Min/+) mice given an isolate of *F. nucleatum* from a patient with inflammatory bowel disease developed gastrointestinal tumors^27–30.^

### The “driver-passenger” model

Tjalsma and colleagues^17^ defined the ‘driver-passenger’ model as intestinal ‘driver’ bacteria with pro-carcinogenic features that potentially initiate CRC development. The colonization of the tumor tissue by bacterial drivers depends on the specific genotype and virulence profile. This is followed by their replacement with ‘passenger’ bacteria, that promote CRC development. Bacterial “passengers” may have metabolic or virulence features that allow them to outcompete the bacterial drivers. The bacterial passengers are often found in patients with advanced disease.

CRC-associated microbiota, such as *S. gallolyticus, E. faecalis, B. fragilis* ETBF, *E. coli*, and *F. nucleatum* can breach and persistently adhere to the colonic mucosa, which may define them as potential “drivers” in the development of CRC. Because their mucosal adherence is likely necessary for their oncogenic potential, this allows them a more intimate contact with the epithelium, affecting the rate of initiation and progression of CRC by promoting inflammation via the stimulation of innate receptors.^31^

A study published in 2014 using pyrosequencing of bacterial 16S rRNA genes from either healthy, adenoma, or tumor biopsy samples from patients were able to identify some bacterial families as “driver” or “passenger”.^32^ They identified seven bacterial genera as potential “driver” bacteria including new potential “drivers” (unclassified *Pseudomonadaceae* and *Neissenaceae*) and pro-inflammatory “passengers” (*Staphylococcus* and *Veillonella*). Their data also identified 12 bacterial genera as potential “passenger” bacteria including the protagonists of tumor development *E. coli* (*Enterobacteriaceae*) and *Streptococcus gallolyticus* (Sgg). However, the enterotoxigenic *Bacteroides fragilis* and *Fusobacterium spp*. strains did not appear in significant abundance across any of the sampled tissues, which could be explained by the small biogeographical diversity of the patient population studied which was limited to the Yunnan Province in China. Another study in 2018 identified that the predominant bacterial phyla associated with familial adenomatous polyposis (FAP) were Bacteroidetes (ETBF) and Firmicutes (*pks+ E. coli*), classifying them as “drivers”.^33^ Further, the study suggests that co-colonization with ETBF and pks+ *E. coli*, promotes carcinogenesis through mucus degradation which enables adherence of more pks+ *E. coli*, inducing increased colonic epithelial cell DNA damage by colibactin and the production of IL-17 by ETBF.

However, there is not a clear picture for what delineates a certain species as a “driver” or as a “passenger” in human CRC cases. Here, are some of the most relevant studies that describe the most well-studied bacteria in CRC and their potential roles in this model.

## Microbiota species most commonly correlated with CRC pathogenesis

Even though the human GI tract harbors a myriad of symbiotic bacteria living in homeostasis with the immune system, some of those can turn into a pathogenic phenotype in susceptible hosts or after exposures to environmental and lifestyle factors and can be considered risk factors for chronic inflammatory diseases like CRC.

### Helicobacter pylori

*Helicobacter pylori*is a small gram-negative bacillus that has been linked to the development of gastric cancer by infecting gastric epithelial cells and producing oxidative stress that modifies the intra-gastric environment.^34^ Two of its virulence factors, the cytotoxin-associated gene A (CagA) and the vacuolating cytotoxin A (VacA), are polymorphic and affect several host cellular pathways.^35^ Even though there is some data to support a potential association between *H. Pylori* and CRC, its association remains controversial. First, in 2006, Zumkeller *et al*. showed a slight increase in risk of CRC in patients with *H. pylori* infection, but this positive association could have been possibility attributed to publication bias and the sources of heterogeneity between studies used in their meta-analysis.^36^ In 2014, a larger meta-analysis of studies conducted in an East Asian population found an increased risk in colonic adenoma development but not in CRC.^37^ As of today, the mechanisms in which *H. pylori* may induce CRC remain hypothetical and need additional investigations.

### Streptococcus bovis/streptococcus gallolyticus (Sgg)

*Streptococcus bovis* biotype I, also known as *Streptococcus gallolyticus (Sgg) is a* gram-positive bacterium belonging to the Group D streptococci. *Sgg* belongs to the *Firmicutes* family, and despite being a commensal of the gut microbiota, it is often associated with septicemia and endocarditis in older people. Interestingly, 25–80% of patients with *Sgg* in the bloodstream appeared to have colon adenomas.^38^The role of *Sgg* in the occurrence of CRC is still controversial, from being either an “opportunistic/passenger” bacteria or a “pathogenic/driver”, which will be discussed further into this review.

### Enterococcus faecalis

Among the species of *Enterococcus*, only two are commonly found in humans: *E. faecalis* and *E. faecium. E. faecalis* belongs to the phylum Firmicutes and is a bacterium that is commonly present in the GI tract (10^5^–10^7^ colony-forming units (CFU)/g) as well as in feces. The frequency is influenced by the host’s geographical location, and especially by their diet. Enterococcus can also be found in other areas of the body, such as the mouth or the vaginal tract, but may cause life-threatening infections if it contaminates a wound, blood, or urine. *E. faecalis* has been associated with CRC pathogenesis due to the production of reactive oxygen species (ROS) that can damage DNA^39^ and create genomic instabilities in the colonic epithelium. Controversially, *E. faecalis* has also been ascribed a protective role by inducing IL-10 and proinflammatory cytokines (e.g. IL-8) in infants^40^ which is why it has been adopted as a probiotic in the treatment of some infant diseases.^40^

### Bacteroides fragilis

*Bacteroides fragilis* is a common gram-negative anaerobic bacterium belonging to the *Bacteroidetes* phylum. *B. fragilis* has two subgroups, the enterotoxigenic strains (ETBF) that possess the *bft* gene encoding the *B. fragilis* toxin (BFT or fragilysin), while the nontoxigenic strains (NTBF) do not. ETBF has been implicated in diarrhea^41^ and is considered oncogenic under certain circumstances due the actions of BFT which can induce DNA damage and inflammation *in vivo*.^18,42^ BFT is a metalloproteinase and has been shown to rapidly alter the structure and function of colonic epithelial cells, including the cleavage of the tumor suppressor protein, E-cadherin. Once cleaved this triggers β-catenin/Wnt signaling leading to permeability of the intestinal barrier. One of the molecular mechanisms in which ETBF triggers colitis and induces colon tumorigenesis in multiple intestinal neoplasia (Min) mice, is via the activation of signal transducer and activator of transcription 3 (STAT3) and induction of T-helper type-17 (T_H_17) T cell responses.

### Escherichia coli

*Escherichia. coli* (*E. coli)* is a gram-negative member of the *Enterobacteriaceae* family. *E. coli* colonizes the intestinal tract of human infants immediately after birth and helps to maintain normal intestinal homeostasis.^43^ Despite its relatively low numbers compared to other commensal bacteria, *E. coli* is a very common cause of intestinal disease.^44^ Several different *E. coli* strains cause diverse intestinal and extraintestinal diseases by means of virulence factors that affect a wide range of cellular processes. The *E. coli* strains are classified as commensal strains (if lacking specialized virulence factors), intestinal pathogenic strains (diarrheagenic), and extraintestinal pathogenic *E. coli* (ExEPEC) strains.^45^ While the diarrheagenic *E. coli* strains have not been linked directly to IBD, they do induce intestinal inflammation causing diarrhea or dysentery. The ExEPEC strain that can causes extra-intestinal infections, including urinary tract infections and meningitis, are the enteropathogenic *E. coli* (EPEC), Shiga toxin-producing *E. coli* (STEC), enterotoxigenic *E. coli* (ETEC), enteroaggregative *E. coli* (EAEC), enteroinvasive *E. coli* (EIEC), and adherent *E. coli* (DAEC).^46^ There are several highly adapted *E. coli* clones that have acquired specific virulence attributes allowing them to adapt to new niches in the gut and cause a broad spectrum of disease. These virulence attributes are frequently encoded on genetic elements that can be mobilized into different strains to create novel combinations of virulence factors. During inflammation, *E. coli* often becomes a dominant member in the gut microbiota. This phenotype is particularly associated with clinical irritable bowel disease (IBD) and, in animal models, with chronic inflammation.^47^ In addition to the observed changes in its abundance, *E. coli* is particularly interesting because it can also alter its gene expression in an inflamed gut. Evidence for changes in gene expression come from the analysis of clinical isolates from patients with a chronic disease, such as IBD and CRC. These studies demonstrate that *E. coli* alters its functional characteristics by inducing a more pathogenic phenotype, including an increase in its adherence and invasive abilities.^48^ A genotoxin encoded by the 54-kb polyketide synthase (PKS) genotoxicity island has been found in *E. coli* isolates from patients with IBD and CRC.^49^ Even gender-specific differences in CRC development has been linked to hemolytic type I *E. coli*, which is significantly associated with adenoma and CRC in female patients only. This was linked to the activation of the expression of the tumor suppressor BIM by acting in part on hypoxia-induced α-subunit.^50^

### Fusobacterium nucleatum

*Fusobacterium nucleatum* belongs to the *Fusobacteria* family and is mostly abundant in the oral cavity where it is associated with dental plaques and periodontal disease. It has also been isolated from patients with CRC and its pro-tumorigenic effects have been verified in experimental models. Like *E. coli*, there seem to be pro-tumorigenic effects due to inflammation caused by the expression of the microorganism’s own genes as evidenced by the presence of *F. nucleatum* and the inflammatory response found in the tumor microenvironment. *F. nucleatum* secretes an anchoring adhesion molecule, FadA, which interacts with E-cadherin on intestinal epithelial cells resulting in an impairment of the barrier and translocation of pathogens. This promotes an inflammatory response and activates β-catenin signaling to further enhance the activity of pro-oncogenic pathways.^27,51^

### Clostridium septicum

Normally found in the gastrointestinal tract, *Clostridium septicum* is a gram-positive spore-forming anaerobic bacillus, that can translocate to the blood triggering bacteremia and gas gangrene, causing up to a 79% mortality rate within 48 hours.^52^ In Addition, 71–85% of patients with *C. septicum* gas gangrene have an underlying malignancy which is most often found in the colon. Even though *C. septicum* does not appear to initiate carcinogenesis, it creates an acidic tumor microenvironment that favors a hypoxic milieu in order to promote spore germination and growth as well as the growth of other pathogens. Its growth in the gut mucosa, is also associated with mucosal ulceration and CRC pathogenesis due to the secretion of its alpha toxin.

### A dual role for streptococcus gallolyticus in the driver-passenger model

The *Streptococcus gallolyticus* (*Sgg*) species has been postulated to be both a “driver” and a “passenger”.^53^ As a driver bacteria, Sgg is found to be predominant in pre-malignant tissue and induces specific inflammatory cytokines (IL-1, COX-2, and IL-8) increasing cell proliferation via the upregulation of the β-catenin pathway and its oncogenic downstream targets (c-Myc and cyclin D). In the passenger model, the presence of *Sgg* in hyperplastic tissue activates the *Wnt* pathway downregulating Slc10A2, a bile acids transporter leading to activation of a specific “bacteriocin” which enables *Sgg* to eliminate commensals like *Enterococci*. Together, these effects of Sgg accelerate transformation from pre-malignant to malignant epithelium.^53^ Another recent study published by Aymeric and colleagues found that in mice genetically prone to CRC colonization by Sgg to be 1,000-fold higher in tumor-bearing mice. The Sgg were found to be secreting a specific “bacteriocin” (called “gallocin”) that can kill closely related gut commensals.^54^ Thus, they concluded that *Sgg* is a cancer-promoting bacterium (a “driver”) only if pre-malignant conditions (in this case genetic susceptibility) exist for *Sgg* to become a driver bacterium, *Sgg* first needs to colonize the colon as a passenger bacterium.

## Physiological functions of CRC-associated bacterial drivers and passengers

### Mucus in driver-passenger model

All that separates our own cells from the trillions of microbes comprising the microbiome is the mucus barrier. The mucus barrier not only creates a physical barrier between us and our own commensals but also protects against many intestinal pathogens, including *Yersinia enterocolitica*,^55^ *Shigella flexneri, Salmonella typhimurium*,^56^ and *Citrobacter rodentium*.^57^ Our own commensals have evolved mechanisms to adhere to the mucus layer and form biofilms, or to use mucus as a source of energy through its digestion.^58^ Disruption or alteration of mucosal integrity is associated with human diseases such as cystic fibrosis and IBD, both of which are also at an increased risk for developing CRC.^18,21^

The intestinal mucus is composed of mucins (MUC2, MUC3 and MUC4) which are aggregates of O-linked glycoproteins (O-glycans) produced by goblet cells^59^ that form a polymeric network of glycosylated proteins. MUC2, the most abundant mucin in the small intestine and colon, can either be secreted to form a gel or produced as membrane-bound glycoproteins that are part of the epithelial glycocalyx. MUC2 is also used as a growth substrate for a distinct subset of anaerobic “driver” species known as mucin-digesting bacteria, including *Akkermansia muciniphila*,^60^ *Bacteroides thetaiotaomicron*,^61^ *Bifidobacterium bifidu*,^60^ *Bacteroides fragilis*,^62^ *Ruminococcus gnavus*,^60^ and *Ruminococcus torques*.^60^ These bacteria digest mucin using specific enzyme glycosidases that release mucin-derived sugars providing a direct source of carbohydrates that promote the growth of both commensal and pathogenic “passenger” bacteria. This is illustrated by *Prevotella* and vancomycin-resistant *Enterococci* who both obtain nutritional support through the degradation of mucus by other commensal species.^63,64^ In addition to nutrional support other CRC-associated bacteria may utilize the sugars produced by mucolytic bacteria to enhance or modify their virulence genes or even impact biofilm formation as seen in the cases of enterohemorrhagic *E. coli* (EHEC) and *adherent E. coli*, respectively.^65^

### Biofilms in the driver-passenger model

Biofilms are formed on the surface of intestinal epithelia and interact with the secreted or membrane-bound mucins, affecting the mucin production related to CRC development.^66^ Mucus-invasive biofilms have been found in the the colon of over half of CRC patients while present in only 13% of healthy subjects. Dejea *et al*. also reported that bacterial biofilm formation is present in 89% of the epithelium of right-sided colon cancer, whereas only 12% is present in left-sided cancers. Metabolomic analysis further confirmed that biofilm formation in the colon is associated with a pro-oncogenic state.^67^

Biofilms are complex communities of microbes that adhere to mucus and allow bacteria normally eliminated to remain within the intestine. While biofilms have been associated with CRC, their presence is not restricted to malignant tissue. In fact, biofilms are present even some distance away from the tumor. Also, the specific combination of bacteria in the proximal colon might be more efficient in forming biofilms. Dejea and colleagues have shown that predominant bacterial phyla associated with adenomas were Bacteroidetes and Firmicutes (family *Lachnospiraceae* including *Clostridium, Ruminococcus*, and *Butyrivibrio*) and some *Fusobacteria* or *Gammaproteobacteria* (especially the *Enterobacteriaceae* family).^68^ Therefore, the organization of bacterial communities into biofilms (higher-order spatial structures of bacterial species) may be necessary for bacteria-induced CRC initiation.^69^ A thinner mucus layer caused by disease, such as the impaired mucin production observed in patients with IBD, can allow bacteria such as *B. fragilis* and *Enterobacteriaceae* to breach this barrier. Once in direct contact with epithelial cells they can form biofilms which may relate to the increased risk of CRC development in this population.^66^ More data suggesting that the composition and organization of the microbiota are associated with CRC tumor development is a study that demonstrated the inoculation of biofilm-positive bacteria originating from either healthy human colon tissue or from a sporadic CRC patient caused the development of tumors in 3 distinct genetic mouse models for CRC (germ-free ApcMinΔ850/+;IL10–/ – mice, the ApcMinΔ850/+ mice or the pathogen-free ApcMinΔ716/+ mice).^70^

**Figure 1.**
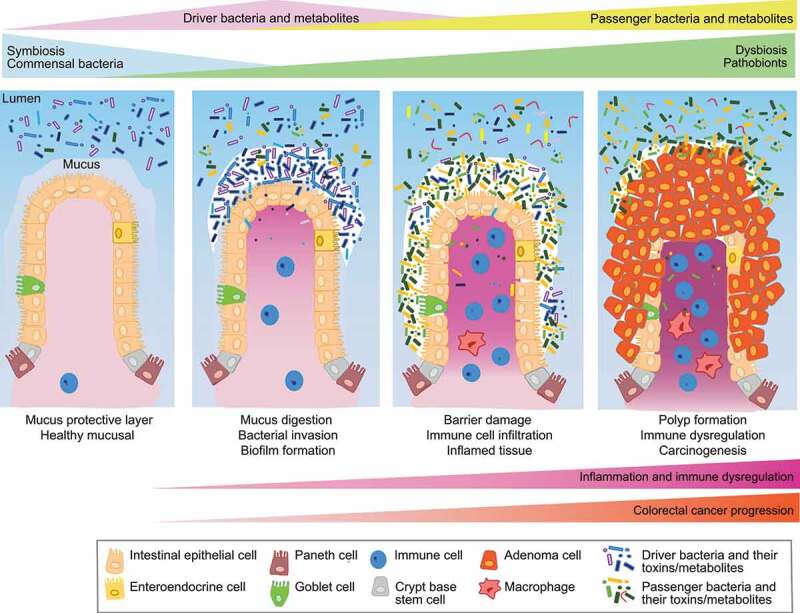
**Implication of the intestinal microbiota “Driver-Passenger” model in CRC**. In the healthy mucosal tissue, many commensal microbes live in symbiosis in the intestinal lumen side of the colon where the intestinal epithelium is protected by a thick layer of mucus. Then, at the beginning stages of colorectal cancer, “driver” bacteria are able to initiate the development of disease via the digestion of the protective mucus layer and the formation of a biofilms. Following the initiation of tumorigenesis an alteration to the microenvironment occurs, resulting in the overgrowth of “passenger” bacteria. This colonization further dysbiosis and leads to the progression of CRC through chronic inflammation and immune dysregulation. In addition, the biosynthesis of toxins directly damaging DNA, and the production of toxic metabolites from the microbiota play an important role in the occurrence of CRC

The intestinal epithelium interacts with the microbiota in a spatially dependent manner therefore, the formation of biofilms may be necessary for “driver” bacteria-induced CRC initiation.^69^ Biofilms are emerging as important factors in tumor initiation and progression; however the heterogeneity observed in the study of biofilms can be due to the heterogeneity of the population sampled, the clinical state of disease or differences in the sampling location. These demonstrate the need for more specific tools and approaches to identify changes to the microbiome and biofilms in gut disorders like CRC. While the mechanism driving the presence of tumor-associated biofilms in the proximal colon is not well understood. Figure 1 illustrates the intestinal mucus and the biofilms formation that might accompany colonization of certain bacteria in proximal of tumor formation, suggesting that there may be a direct mechanistic link with mucinous CRC.

### Gut microbiota metabolites as “passenger-precursors” in promoting CRC

The human body is continuously exposed to dietary compounds of microbiota, the resident and transient microbiota, as well as their secreted products including toxic metabolites that interact with gut epithelial cells at the mucosal surface.^19^ Changes of the local metabolic environment in the gut may impair the mucosal barrier, allowing metabolites to diffuse into the intestinal lumen. For example, *E. faecalis* produce extracellular superoxide which can induce DNA damage or cause chromosomal instability, resulting in malignant transformation of mammalian cells. In addition to their own endogenous metabolites, the intestinal microbiota can also convert ingested nutrients into metabolites that target other microbes, host cells or both. Therefore, metabolites would act as small messengers between the intestinal microbiota and human cells, to modulate immune and epithelial cell functions. This crosstalk between dietary or bacterial metabolites and immune cells may partially explain a dietary basis for inflammatory diseases.^2^

**Figure 2.**
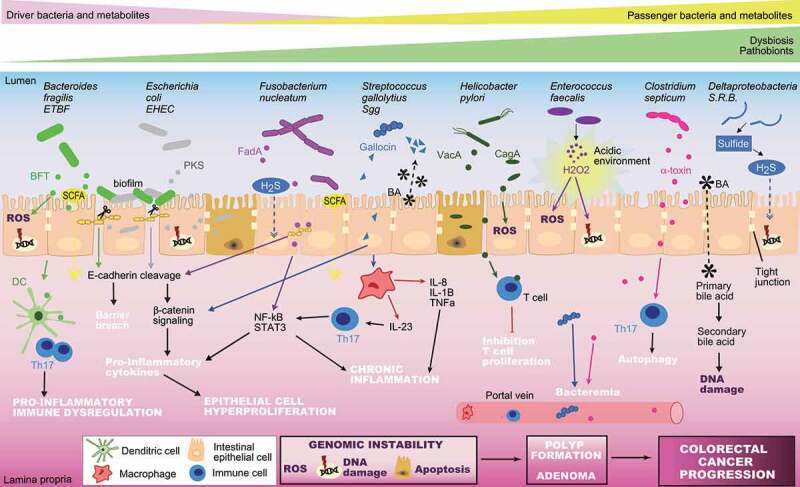
**Principal Driver-Passenger microbes associated with colorectal cancer**. These are the main microbial Driver and Passenger during multistep colorectal cancer progression. This schematic summarizes the major microbes and their genotoxin and/or metabolites that may cause alterations to the epithelium and the mucosa during CRC. BFT, Bacteroides fragilis toxin; PKS, polyketide synthase (colibactin); FadA, Fusobacterium adhesin; CagA, cytotoxin-associated gene A; VacA, vacuolating cytotoxin A; BA, Bile acid; SCFA, Short-chain fatty acids; ROS, reactive oxygen species; STAT3, signal transducer and activator of transcription 3; H2S, hydrogen sulfide

According to the “driver-passenger” model, one can speculate that “drivers”- and their corresponding metabolites may cause DNA damage and promote the malignant transformation of epithelial cells. Once tumorigenesis is initiated, this process promotes a change in metabolite composition and reduced mucosal barrier allowing the opportunistic pathogens or “passengers” to colonize tumor sites. An overview of four of the main metabolites and their role in the “driver-passenger” model is addressed below and summarized in Figure 2.

### Short-chain fatty acids (SCFAs)

Short-chain fatty acids (SCFAs) are produced via the fermentation of dietary components, such as indigestible fibers in the proximal colon. SCFAs are defined as saturated fatty acids displaying up to six carbon residues and are known as acetic (C2), propionic (C3), and butyric (C4) acids.^71^ To produce SCFAs, it is important that the gut microbiota work as a community, but also have symbiotic associations with the host. For instance, saccharolytic members of Bacteroidetes are the primary fermenters of nondigestible carbohydrates in the proximal colon producing mainly produce acetate and propionate.^71^ In contrast, butyrate is the metabolic end-product of members of Firmicutes. Of the different microbial metabolites that have the potential to promote and protect against colorectal cancer, SCFAs are among the most notable and well-studied.^2^ Accumulating evidence suggests that microbial-derived SCFAs impact T regulatory T cell populations through the inhibition of host histone deacetylases and interactions with cell surface receptors.^72^

### Hydrogen sulfide (H2S)

Initially considered as a cysteine-derived endogenous gas, hydrogen sulfide (H2S) is a labile, diffusible gas, with a bell-shaped dose–response curve. While H2S is the most studied sulfur derivative and is now recognized as a key regulator of gut health,^73^ the extent to which it is damaging or beneficial remains unclear. This is due to the pleiotropic effect H2S has on the intestinal epithelium whereas it is protective effect at low concentration, H2S has detrimental effects at higher concentrations. Elevated levels of H2S was first identified in patients with colon cancer in 1996^74^ and both endogenous and microbial H2S production from cysteine were upregulated at the tumor site in colorectal cancer patients.^75^ Due to its ability to inhibit cellular respiration,^76^ H2S is considered to be a causative metabolite in CRC. Several indications point to sulfate-reducing bacteria (SRB) as potential players in the etiology of intestinal disorders due to their ability to produce H2S, even though their role as “driver” or as “passenger” remains unclear.^77^

Of all the microbes in the intestine, only SRB rely on inorganic sulfate for conservation of energy. Through a type of anaerobic respiration,^78^ SRB oxidize molecular hydrogen (H2) while reducing sulfate (SO4^2–^) to create hydrogen sulfide (H2S). Diets supplemented with organic sulfur-compounds (cysteine, taurocholic acid, and mucin) (mostly found in red meat, eggs and milk) provide a more efficient source for H2S production within the colon^79^ than inorganic sulfate (such as breads, dried fruits, vegetables, nuts, fermented beverages). More than 25 genera of SRB belong to the *Deltaproteobacteria* family, while others belong to Clostridia *(Desulfotomaculum, Desulfosporosinus*, and *Desulfosporomusa genera)*.^77,80,81^ Since *Desulfovibrio* bacteria seem to be ubiquitous in the bowel, but heavily colonized in disease, they are most likely to play a role in pathogenicity of gut inflammation possible linked with the sulfide detoxication pathways or bacterial antigen presentation.

Recent studies have identified SRB as new bacterial “drivers” of colitis and/or tumorigenesis in preclinical models. The presence of *Desulfovibrio* subspecies which produce toxic sulfide was shown to be increased at multiple sites within the colon of ulcerative colitis patients compared to healthy controls.^82,83^ Further, Mottawea et al.^84^ showed that abundance of H2S-producing bacteria, including *Atopobium parvulum*, was positively correlated with pediatric IBD disease severity. It is likely that *Atopobium parvulum* produces H2S during the fermentation of sulfate-containing compounds^27,28,51,85^ as suggested by a reduction in carcinogenesis by treatment with bismuth, a H2S scavenger in an IL-10-/- model of CRC.^84^ Tsoi et al^86^ demonstrated that ROS and cholesterol produced by *Peptostreptococcus anaerobius*, which is increased in human colon tumors, enhances AOM-induced tumorigenesis in mice through TLR2/4. Like A. parvulum, *F. nucleatum* also ferments methionine to produce H2S and was recently shown to enhance colonic tumor development in the Apc min/+ mouse model, suggesting a potential role of *F. nucleatum* as “driver” in the etiology of CRC.^87^

### Reactive oxygen intermediates (ROS)

Reactive oxygen intermediates (ROS) are chemically reactive molecules containing oxygen. ROS such as superoxide, hydrogen peroxide, hypochlorous acid, singlet oxygen and hydroxyl-free radicals have been linked to cancer because of the important roles they play in several inflammatory pathways (e.g., NF-KB, ERK1/2, p38, PI3K, and others) and their ability to induce oxidative DNA damage.^88^ Most ROS is generated in the mitochondria as by-products of physiological processes that utilize ROS-generating enzyme or by interactions with potentially harmful exogenous factors.^89^ Interestingly, changes in the microbiota correlate with changes in mitochondrial metabolism and mitochondrial DNA.^90^

A number of commensal species associated with CRC also produce high amounts of ROS. For instance, *E. faecalis* produce extracellular superoxide (O(-)(2)), H_2_O_2_ and hydroxyl radical, which can enhance levels of ROS that can damage DNA^39^and enhances the expression of COX-2 in macrophages, promoting chromosomal instability.^91^ The bacterial enterotoxin of *B. fragilis*, BFT, produces spermidine and H_2_O_2_ as by-products of polyamine catabolism^92,93^ resulting in apoptosis, DNA damage, and inflammation. A number of studies have demonstrated a link between *H. pylori* CagA and gastric cancer development.^94,95^ One potential mechanism proposed for this association is ROS accumulation and Akt activation when CagA is degraded by autophagy induced by the *H. pylori* vacuolating cytotoxin, VacA. However, it is unknown if CagA promotes cancer development or if it was coincidentally present in the patient’s serum without causing associated inflammation, despite a study that found an association between CagA seropositivity and increased risk (10.6 fold) in gastric and colonic cancer compared to CagA negative strains.^96^ *Citrobacter rodentium* is often used as an enteric bacterial pathogen to study enterohemorrhagic *E. coli* (EHEC) infections in humans because of the similar inflammatory responses induced.^97^ In 2014, Lupfer et al^98^ showed both in mice and in macrophages a key role of NOD2 and RIP2 in the ROS homeostasis^98^ in a mouse model of enteropathogenic *C. rodentium* infection by demonstrating that ROS regulated caspase-11 expression and non-canonical NLRP3 inflammasome activation through the JNK pathway.

Inflammation is also responsible for site-specific changes in CRC including shifts in pH or oxygen concentration like hypoxia-inducing acidosis.^99^ Interestingly, the gut-brain axis is increasingly recognized as an important pathway of communication where gut microbiota seems to play a significant role in this mutual relationship. For example, Oxidative Stress (OS) is also a key player in the pathogenesis of neurodegenerative diseases, such as Alzheimer’s or Parkinson’s, and acute conditions, such as stroke or traumatic brain injury.^89^

### Bile acids

Bile acids (BAs) are classified into three groups: primary, secondary, and tertiary. The Primary bile acids found in the human gut are cholic acid (CA) and chenodeoxycholic acid (CDCA), which are originally synthesized from cholesterol in the liver and play an important role in fat metabolism.^100^ The secondary BAs are the product of intestinal bacterial metabolism of the primary bile acids, which are lithocholic acid (LCA), deoxycholic acid (DCA) and ursodeoxycholic acid (UDCA).^100^ The tertiary BAs are formed via hepatocyte metabolism of reabsorbed primary BAs, such as the glycocholic acid (GCA) or the taurocholic acid (TCA).^100^ In addition, bile acids can serve as signaling molecules, capable of activating receptors as well as signaling pathways.

Gut bacteria contribute to the concentration of secondary BA in the gut through the metabolism of primary BAs and high levels of secondary bile acids have been correlated with an increased risk of colon cancer.^101^ Secondary bile acids, especially deoxycholic acid (DCA), cause DNA damage of the epithelium leading to apoptosis in a p53-independent manner.^102^ A model linking early CRC development and increased luminal concentration of secondary BAs was described in 2018 by Pasquereau et al.^53^ Here, the “passenger” bacteria *Sgg* activate the Wnt pathway, an early step in CRC development, causing a downregulation of the BA transporter Slc10A2 resulting in the accumulation of BA.

Even though BAs have emerged as important and pleotropic signaling metabolites involved in the regulation of metabolism and inflammation, their direct interactions with both microbiota and host receptors remain unknown. The dynamic three-dimensional interplay between BAs, the microbiome and the mucosal immune system have proven an important area of new drug discovery.

## Profiling microbial metabolites

A better understanding between the human microbiome and different diseases has been possible through the development of culture-independent next-generation sequencing technologies and analyses. However, the function and metabolic niche requirements of bacterial communities in diseases, such as CRC, are largely unknown.

16S rRNA sequencing and next-generation sequencing (NGS) have allowed a deeper understanding of the human microbiome in healthy and disease states. In a culture-independent manner these techniques can identify the variation in diversity and abundance of bacteria in a sample. Amplification of 16S rRNA hypervariable regions can be used to detect microbial communities in a sample typically down to the genus and sometimes the species levels due to its high conservation between bacteria and archaea. Indeed, the NGS, which includes techniques such as whole-genome sequencing and shotgun metagenomics allows taxonomic profiling down to the strain-level detection of particular species. It also allows functional characterization of microbes or microbial communities which is a very valuable tool when trying to link the microbiome to diseases development.^103^

NGS has allowed the growth of a number of multi-‘omics’ technologies^104^ including metagenomics, metatranscriptomics, proteomics, and metabolomics. The latter helps to identify and quantify the metabolites present in a sample, and has emerged as the most suitable method to study the microbiome.^105^ Multi-omics technologies have the potential to predict disease-relevant mechanisms of host–microbial interactions by identifying transcriptional alterations and metabolic changes. For example, metagenomics is limited to revealing the functional potential of microorganisms, rather than the actual functional activity. The presence of a gene or pathway does not necessarily mean that it is expressed. In addition to isolating DNA for metagenomics, RNA can be extracted from a sample, reversed transcribed into cDNA and sequenced for metatranscriptomics that measures actual microbial gene expression. It is important when measuring actual gene expression to also determine the functional potential inferred from gene presence for understanding disease-related microbiota changes. Microbial transcriptional programs can respond rapidly to environmental cues such as changes in inflammation and oxygen levels, which may not necessarily be reflected on the DNA level. However, some caveats apply to fecal metatranscriptomics, such as variation due to subject-specific transit times, and that it only captures extractable, non-degraded RNA restricted to organisms that are present in stool. Although it does not answer questions about gene expression, this approach cleverly enables one to determine which bacteria are actively dividing and presumably transcribing their genes.

These genomic and metabolomic technologies are also facilitating strain-level analysis of the gut microbiome in a variety of diseases. Metabolomics studies of blood samples are used to predict the gut microbiota a-diversity that could lead the way for development of clinical tests for monitoring gut microbial health. For example, Wilmanski and colleagues^106^ have identified a subset of 40 plasma metabolites (over 1000) of which 13 were microbial in origin, as a way to predict metabolic perturbations linked with cardiovascular disease, diabetes, kidney function or extreme obesity. Those metabolite biomarkers could predict the a-diversity in the human host and therefore shape a better diagnostic or therapeutic approaches. Recently, Garza et al,^107^ published an *in silico* computational approach to identify colorectal cancer metabolic passengers. Their model explains the association of bacterial passengers to CRC as being driven by the availability of specific CRC metabolites. They found that metabolic networks of bacteria that are significantly enriched in CRC metagenomic samples either, depend on metabolites that are more abundant in CRC samples, or specifically benefit from these metabolites for biomass production. Therefore, their computational experiments suggest that metabolic alterations in the cancer environment are a major component in shaping the CRC microbiome.^107^

## The intestinal microenvironment linked to CRC

Even though the models described above could explain the alterations in the tumor environment by the oncogenic bacteria allowing opportunistic pathogens to colonize tumor sites, those models have still some limitations. Those models do not explain the variations observed between patients with CRC such as the expression of the *bft* gene in some patients and not others.^108^ Also, others factors like the inherited genetic susceptibility, tabaco or alcohol consumption or dietary components,^109^ or diseases like obesity, diabetes, or chronic gut inflammation are not taken into consideration in those models. As an example, the consumption of probiotics like *Lactobacillus* and *Bifidobacterium* can improve the secretion of the SCFA by the epithelium limiting angiogenesis and therefore the growth of CRC.^110^ Hence, probiotics could decrease the abundance of *Bifidobacterium, Clostridium, Faecalibacterium*, and *Roseburia* bacteria observed in CRC patients and leverage the colonization of commensal bacteria such as *E. coli, E. faecalis, F. nucleatum*, and *S. gallolyticus*. In 2018, Rothschild et al. looked at the association between the gut microbiota and environmental and genetic factors and found that the gut microbiota was predominantly (20% of the variance in microbiome β-diversity) established and influenced by environmental factors like diet or lifestyle rather than genetically heritable (only 1.9%).^111^

It is evident that CRC is no longer associated with a single microbial species or mechanism but rather with the microenvironmental effects of the microbiome community, in which the “driver-passenger” model helps to define the chronologic bacterial changes around the tumor site. Recently, Gaza and colleagues evaluated by metagenomic studies, over 1500 genome-based metabolic models CRC. They found the association of specific metabolites from passenger bacteria to be linked with CRC as well as metabolites from bacteria are more important for biomass production.^107^ Therefore, the combination of CRC-associated metabolites confers a “specific growth advantage” for bacterial genera that profit from CRC derived metabolites.^107^

## Conclusion

Through coevolution there is now a reliance on “commensal” bacteria for coevolutionand the production of metabolites and certain vitamins. However, what constitutes a healthy microbiota has not been identified. Certain intestinal bacterial may contribute to mutations that occur during cancer development in the gut.^32^ Tjalsma *et al*.^17^ have proposed a driver-passenger model to explain the involvement of specific bacteria in the origin and/or proliferation of CRC. Under this model, both driver and passenger bacteria would play distinct roles in eliciting the gut epithelium transformation from normal to hyperplasia, and adenoma to malign carcinoma.
